# New York Veterinary Colleges

**Published:** 1896-04

**Authors:** 


					﻿NEW YORK COLLEGE OF VETERINARY SURGEONS.
The session closed on April ist. The exercises were an innovation, con-
sisting of a conferring of the degrees in connection with a banquet. A more
complete report will appear in the May number.
				

